# The Oculist's Vade-Mecum: A Complete Practical System of Ophthalmic Surgery

**Published:** 1844-01

**Authors:** 


### ART. XVIII.

*The Oculist's Vade-mecum: a complete Practical System of Ophthalmic Surgery.* By JOHN WALKER.—*London,* 1843. 12mo, pp. 400. One coloured plate, and Twenty Woodcuts.

THAT to make a book on the diseases of the eye, and even a long and a tedious one, is no difficult task, is sufficiently proved by the many ponderous volumes on that subject which load the shelves of our medical libraries. To give a novel and succinct account of this class of maladies in a perspicuous and readable style, is not so easy: we think Mr. Walker capable of doing this; but we are sorry that this has not been so much his object in the present work, as that of abridging the productions of his laborious predecessors. The work he has undertaken to do, however, he has accomplished with considerable success.

We cannot flatter Mr. Walker as to the "pictorial illustrations," as he is pleased to call them, which accompany his book. He hopes that they will "add materially to the value of the work." We think they had much better been omitted. They must add to the price, while they appear to us for the most part of little or no use.

"It forms no part of the object of this treatise," says Mr. Walker, "to enter into abstruse disquisitions on the nature of those actions which give rise to the phenomena of ocular disease, but rather the more practical one of describing, as accurately as possible, the phenomena themselves, their causes, and, more especially, the treatment they require." (p. 2.)

In pursuing this object, Mr. Walker gives a detailed account of what is most important in each of these topics, in relation to the various diseases of each individual structure of the eye, commencing with those which are external, viz. the conjunctiva, cornea, and sclerotica; and afterwards proceeding to those deeply seated; the iris, the choroid, and the retina. Then follows the consideration of the affections of the aqueous, crystalline, and vitreous humours, and their capsules. He next enters on the subject of those diseased conditions which simultaneously involved the whole, as well as those few anomalous affections, which, from the uncertainty as to what parts they really reside in, could not properly be classed with the preceding. Lastly, the morbid state of the appendages claim our author's attention.

The nature of the work does not claim an extended or critical notice from us; we will, however, advert to a few particulars which we noted for comment during perusal.

We cannot admit that Mr. Walker has described any specific disease of the eye under the head of "simple ophthalmia." Most of the cases which he would class under this name are, we are persuaded, nothing else than instances of catarrhal ophthalmia, while others of them are examples of the sympathetic redness of the conjunctiva, attending inflammation of the deeper textures, such as the iris. The case related in page 11, we regard as one of iritis, attended with conjunctival redness. The case in page 18, appears to have been scrofulous ophthalmia. Pustular ophthalmia, which is surely a very different disease from either catarrhal or scrofulous ophthalmia, differs but little, Mr. Walker tells us (p. 63) from his "simple ophthalmia." Such is the confusion arising from admitting into a nosological arrangement, a disease which has no specific character of its own, but merely borrows an existence, so to speak, from a group of others.

Among the remedies mentioned by Mr. Walker, we are sorry to observe sugar of lead (p. 17), a substance, which, on account of the deplorable effects it produces when there happens to be any degree of ulceration of the cornea present, we had believed to be discarded from ophthalmic practice.

Mr. Walker is extremely fond of the solid nitrate of silver. He seems to have recourse to it, even in the most trifling cases of inflammation of the eye. For example, in pustular ophthalmia, (p. 66,) he touches the inside of the lower lid with the caustic, a piece of practice we should deem quite superfluous. In ulcer of the cornea, also, he applies the same remedy to the conjunctival surface of the lower lid. (p. 124.) He tells us (p. 19) that the use of this favorite application sometimes produces ulceration of the conjunctiva, and even that "inversion of the lid results from it." Now, if such consequences arise in the cautious and experienced hands of Mr. Walker, assuredly in those of ordinary practitioners, for whom the work before us is in a great measure intended, the remedy would prove, in general, ten times more dangerous than some of the slight affections which Mr. Walker recommends to be treated with it.

Mr. Walker's description of purulent ophthalmia is good, and the discussion into which he enters on the various points of treatment is shrewd and interesting. We think he estimates antiphlogistic treatment too low, and trusts too exclusively to stimulants. He utterly repudiates bleeding, (p. 36,) as likely to favour sloughing of the cornea, and regards the solid nitrate of silver as the sheet-anchor in the treatment of purulent ophthalmia. He applies it freely to the conjunctiva for a few seconds, once a day, insinuating it beneath the margin of each lid. If a sloughing condition of the cornea seems impending, Mr. Walker recommends a generous diet, wine and quinine. The case of Mr. A. (p. 52) is instructive. The treatment was commenced on the second day of an attack of gonorrheal ophthalmia; all the symptoms were of an aggravated character; ulceration of the cornea was already apparent; chemosis was developed to the fullest extent, and the tension of the lids was excessive. A cure was accomplished without the abstraction of a drop of blood, and with no other local applications than the lunar-caustic pencil to the conjunctiva, and warm water to the cutaneous surface of the lids.

It is well known, that after the secondary fever of smallpox, inflammation is apt to attack the eye, and to destroy it. Mr. Walker thinks (p. 82,) that this occurs under the form of severe catarrhal or purulent ophthalmia, which most assuredly is not the case. The form under which it occurs is that of abscess within the substance of the cornea, ending in extensive ulceration. His plan of treatment, applying nitrate of silver freely to the conjunctival surface, would probably put out the eye. He does not seem to have tried it, and we hope he never will.

Mr. Walker makes a laboured attempt (pp. 89, 90,) to explain the triangular shape of pterygium, but leaves the matter no clearer than he found it. It is evidently often the case that this disease arises from the existence of an elevated point, at the edge, or on the surfaces of the cornea, to which, as a cause of irritation, the enlarged vessels of the conjunctiva are attracted. We have our doubts, whether some such cause is not always in existence. If it is so, the triangular form of the enlarged vessels ceases to be at all mysterious.

For the relief of the imperfect vision which results from conical cornea, Sir W. Adams proposed a removal of the lens, and some one referred to, but not named, by Mr. Middlemore, has advised an artificial pupil. Mr. Walker has combined these two plans, first making an artificial pupil behind the edge of the cornea, and then extracting or dividing the lens. He relates an interesting case, in which the result of the operative proceedings was highly satisfactory, so far as the first eye was concerned. In the second, the artificial pupil was successfully formed, but inflammation followed the extraction of the lens, and destroyed the eye. (p. 145.) In the second eye, it would have been satisfactory to have tried extraction before forming the artificial pupil, as putting Sir W. Adam's plan to the test. Had the eye recovered from the extraction, but vision remained as before, the artificial pupil might have been formed.

At page 158, Mr. Walker states that thickening or deposition never affects the sclerotica, in consequence of inflammation. The reverse is the fact; a thickened and fleshy, and sometimes a thickened and pearly appearance, is a frequent result of the disease termed sclerotico-choroiditis, the same disease which in a more advanced stage gives rise to attenuation of the sclerotica, and protrusion of the choroid.

Speaking of staphyloma scleroticæ, Mr. Walker says, it is necessary to excise the whole cornea, or at least a considerable part of it, and, in some cases, the projecting portion of the sclerotica also, to effect the sinking of the eye. (p. 166.) Now, when the staphyloma surrounds the whole cornea, and the eye is greatly enlarged, no doubt the anterior third of the eye, or thereabouts, including the cornea, must be removed; but if the staphyloma is confined to one side of the eye, it is sufficient to remove the part affected, leaving the cornea untouched.

The figure of coloboma iridis given in page 208, is incorrect. The natural pupil always exists to the full extent in that malformation, and added to it is a prolonged slit, generally downwards, but sometimes to one side. Mr. Walker's figure represents the natural pupil as in a great measure wanting.

The old-fashioned question about the proper season for operating for cataract has lately been revived in this country. The following is Mr. Walker's opinion upon this point:

"Some writers insist that an operation for extraction of cataract ought not to be undertaken during the winter season. I confess that I see nothing in the objection. If the patient be in good health at the time, I should say that there is nothing adverse in the season of winter. A more reasonable objection might be urged against the great heat and bright sunlight of summer, which are more difficult to guard against than the cold of winter. My own experience leads me to conclude that the operation is at least as successful in winter as in summer. In this country, where usually the winters are mild, and the seasons temperate, the periods are few and of short continuance in which the operation would be improper. Of the two, I should certainly more strongly object to the summer, but I should also decline operating in a very frosty season." (p. 252.)

Mr. Walker gives five cases of extraction of cataract, (p. 260,) which will be read with interest even by those who have operated much, and ought to be studied attentively by those who have not yet operated. The following remarks on the unfavorable results of the operation of division, we deem important.

"A not unfrequent termination of the operation of division and the subsequent absorption of the cataract and one which I conceive has not been sufficiently pointed out, is that of atrophy of the eye. It would seem that the process of absorption is not always limited to the cataract, but occasionally extends to the vitreous humour and the other structures of the globe. I have seen many instances of such a result, not confined, indeed, to the operation for cataract, but likewise in cases where the lens had been injured by sharp instruments accidentally passed into the eye. Nor is it to be wondered at that the repeated introduction of an instrument within the interior of the eye, a proceeding which must inevitably break up the delicate cells of the vitreous body, together with the irritation consequent thereupon, and on the presence of the disorganized lens, combined with an unnatural excitement of the absorbent vessels, kept up for many weeks or months,—should be succeeded by a much greater amount of absorption than was originally contemplated, and that ultimately a large portion of the fluid contents of the globe should disappear. The wonder is, that so much can be done in the way of interference with the vitreous body, during the various operative proceedings on the eye, with such frequent impunity." (pp. 277-8.)

Hydrophthalmia between the conjunctiva and sclerotica is, as far as we recollect, a disease hitherto undescribed :

"A labouring man presented himself at the hospital, in whom there was a considerable quantity of fluid collected between the conjunctiva and sclerotica, forming a tumour of some size all around the front of the globe, constituting a case of *hydrophthalmia externa.* In this instance the eye had been previously lost by an accident, the cornea had become opaque, and the eye-ball much shrunk; but the presence of this watery swelling gave to the eye the appearance of being more prominent than natural. The fluid, which was contained in three or four separate compartments, apparently communicating with each other, was evacuated by a puncture, but speedily collected again; afterwards the conjunctiva was dissected off, and no further secretion of the fluid took place." (p. 310.)

Opening the cornea by a large incision, removing a portion of it, and afterwards poulticing the eye, as practised by Mr. Barton, appears to answer in cases of pieces of percussion-caps lodging within the eye. The foreign body escapes, and the irritation ceases. Of this, Mr. Walker gives an illustration at page 323. The extension of the practice to cases of lacerated eye, producing sympathetic retinitis of the opposite eye, does not appear so likely to do good. It failed in a case related by Mr. Walker at page 328. "Its failure," observes our author, "was probably to be attributed to the advanced state of the disease, and to the circumstance of the operation not having been resorted to at a sufficiently early period."

If, in the above brief remarks, we have mingled a large proportion of criticism, we can assure Mr. Walker it is from no desire to depreciate the value of his work, which, as a whole, does him a great deal of credit, and will undoubtedly prove, to many, a useful introduction to the study of the diseases of the eye.

